# Alteco endotoxin hemoadsorption in Gram-negative septic shock patients

**DOI:** 10.1186/cc12006

**Published:** 2013-03-19

**Authors:** HP Shum, KC Chan, WW Yan

**Affiliations:** 1 Pamela Youde Nethersole Eastern Hospital, Hong Kong

## Introduction

Septic shock is a common cause of mortality and morbidity in the ICU. Endotoxin hemoadsorption using a Polymyxin B fiber column can improve patient outcome [1]. This study investigated the therapeutic effect of a new endotoxin hemoadsorption device in Gram-negative septic shock patients.

## Methods

An open, controlled, prospective, randomized, single-centre trial conducted between February 2010 and June 2012. Patients with septic shock due to intra-abdominal sepsis were recruited and randomized to either standard therapy (ST, *n *= 8) or standard therapy plus two 2 hourly sessions of Alteco endotoxin hemoperfusion (AT, *n *= 7). Standard therapy included infective source control, appropriate early antibiotic, low-dose steroid, early continuous renal replacement therapy in the presence of acute kidney injury (RIFLE Injury class or more), hemodynamic optimization and lung-protected ventilatory support. Primary outcome was change in organ dysfunction at 48 hours measured by Sequential Organ Failure Assessment (SOFA) scores. Secondary outcomes were change in vasopressor requirement (measured by vasopressor score), PaO_2_/FiO_2 _(fraction of inspired oxygen) ratio, total urine output at 72 hours and 28-day mortality.

## Results

This study was terminated early as interim analysis identified no significant clinical benefit. Baseline characteristics (age/APACHE IV score) were similar between two groups of patients. No significant difference was noted between two groups with respect to change in total SOFA score (+1 vs. -5.5 for AT vs. ST, *P *= 0.382), vasopressor score (-29 vs. -46.6, 3= 0.775), PaO_2_/FiO_2 _*P *ratio (-26 vs. +163, *P *= 0.199), total urine output from 0 to 72 hours (3,850 ml vs. 4,570 ml, *P *= 0.355) and 28-day mortality (14.3% vs. 37.5%, *P *= 0.569). No significant side effect was noted when using this new hemoadsorption device.

## Conclusion

This small study cannot identify any extra clinical benefit on addition of Alteco endotoxin hemoadsorption to standard therapy in patients suffering from intra-abdominal sepsis with shock due to Gram-negative bacterial infection.

## References

[B1] Cruz DN JAMA 2009 301 2445 2452 10.1001/jama.2009.856 19531784

